# A survey on the spine fracture epidemiology and risk factors among patients referred to a specialized trauma center in Iran during 1998-2000

**Published:** 2012-11

**Authors:** Hamid Reza Saeidiborojeni, Reza Fattahian

**Affiliations:** ^*a*^Department of Neurosurgery, Kermanshah University of Medical Sciences, Kermanshah, Iran.

## Abstract

**Background:**

Spine injury is among damages that may cause a lot of disabilities and even lead to death. According to international statistics, about 2% to 6% of all trauma cases are related to spine injuries. The incidence of spine injuries may be very different in different countries. Despite the extensive studies in developed countries, there is not enough information on spine injuries in Third World Countries, including Iran. The present study has been designed to examine the patients referred to the Taleghani Hospital in Kermanshah (Iran) which is the main trauma center in the west of Iran.

**Methods:**

This was a descriptive analytical study. Data on all patients with spinal injury diagnosis referred to Taleghani Hospital in Kermanshah was extracted from patient records. Information on age, gender, occupation, education level, anatomic location of injury, accident type and geographic location of the incident were entered into SPSS software. Data were analyzed using descriptive statistics methods.

**Results:**

The statistical population of the study consisted of 300 persons, of which 170 were male and the rest were female. The mean age of men and women was 40 ± 12 and 50 ± 10 years, respectively. In total, 35.4% of patients were housewives, 15.3% unemployed, 14.1% employees and others had other jobs. In total, 188 cases of spine injuries were because of falling, 92 cases because of car accidents, and 20 cases were because of street fights. In this study, 160 cases suffered from the lumbar vertebrae fracture, 82 cases suffered from thoracic vertebrae fracture and 58 cases had cervical vertebrae fracture. In total, 76% of the damage occurred in the urban areas and the rest were in rural areas.

**Conclusions:**

Considering the increasing trend of accidents, especially road accidents in Iran, it seems that the number of spine fracture cases had a progressive trend in recent years. Given the high rate of disability and death in such cases, it seems that more attention must be paid toward preventive measures for all spinal injuries levels.

**Keywords:**

Spine, Fracture, Epidemiology, Trauma


					Volume 4, Suppl. 1 Nov 2012
Publisher: Kermanshah University of Medical Sciences
URL: http://www.jivresearch.org
Copyright:
Congress of Iranian Neurosurgeons, 14-16 November 2012, Kermanshah, Iran
Paper No. 33
A survey on the spine fracture epidemiology and risk factors
among patients referred to a specialized trauma center in Iran
during 1998-2000
Hamid Reza Saeidiborojeni a,*
, Reza Fattahian a
a
Department of Neurosurgery, Kermanshah University of Medical Sciences, Kermanshah, Iran.
Abstract:
Background: Spine injury is among damages that may cause a lot of disabilities and even lead to death. According
to international statistics, about 2% to 6% of all trauma cases are related to spine injuries. The incidence of spine
injuries may be very different in different countries. Despite the extensive studies in developed countries, there is not
enough information on spine injuries in Third World Countries, including Iran. The present study has been designed to
examine the patients referred to the Taleghani Hospital in Kermanshah (Iran) which is the main trauma center in the
west of Iran.
Methods: This was a descriptive analytical study. Data on all patients with spinal injury diagnosis referred to
Taleghani Hospital in Kermanshah was extracted from patient records. Information on age, gender, occupation,
education level, anatomic location of injury, accident type and geographic location of the incident were entered into
SPSS software. Data were analyzed using descriptive statistics methods.
Results: The statistical population of the study consisted of 300 persons, of which 170 were male and the rest were
female. The mean age of men and women was 40  12 and 50  10 years, respectively. In total, 35.4% of patients
were housewives, 15.3% unemployed, 14.1% employees and others had other jobs. In total, 188 cases of spine
injuries were because of falling, 92 cases because of car accidents, and 20 cases were because of street fights. In
this study, 160 cases suffered from the lumbar vertebrae fracture, 82 cases suffered from thoracic vertebrae
fracture and 58 cases had cervical vertebrae fracture. In total, 76% of the damage occurred in the urban areas and
the rest were in rural areas.
Conclusions: Considering the increasing trend of accidents, especially road accidents in Iran, it seems that the
number of spine fracture cases had a progressive trend in recent years. Given the high rate of disability and death
in such cases, it seems that more attention must be paid toward preventive measures for all spinal injuries levels.
Keywords:
Spine, Fracture, Epidemiology, Trauma
* Corresponding Author at:
Hamid Reza Saeidiborojeni: Associate Professor of Neurosurgery, Kermanshah University of Medical Sciences, Imam Reza Hospital, Kermanshah, Iran.
Tel: 09181321399, Email: dr.hamidrezasaeidi@yahoo.com, (Saeidiborojeni HR.).

				

